# Mining Suicidal Ideation in Chinese Social Media: A Dual-Channel Deep Learning Model with Information Gain Optimization

**DOI:** 10.3390/e27020116

**Published:** 2025-01-24

**Authors:** Xiuyang Meng, Xiaohui Cui, Yue Zhang, Shiyi Wang, Chunling Wang, Mairui Li, Jingran Yang

**Affiliations:** 1 School of Information Science and Technology, Beijing Forestry University, Beijing 100083, China; xiuyangmeng@bjfu.edu.cn (X.M.); cuixiaohui@bjfu.edu.cn (X.C.); zhangyue2@bjfu.edu.cn (Y.Z.); limairui@bjfu.edu.cn (M.L.); jranyang@bjfu.edu.cn (J.Y.); 2 Engineering Research Center for Forestry–Oriented Intelligent Information Processing of National Forestry and Grassland Administration, Beijing 100083, China; 3 School of Information Engineering, Minzu University of China, Beijing 100081, China; 22012646@muc.edu.cn

**Keywords:** suicide ideation detection, social media analysis, information gain, dual-channel model, deep learning networks, entropy measurement

## Abstract

The timely identification of suicidal ideation on social media is pivotal for global suicide prevention efforts. Addressing the challenges posed by the unstructured nature of social media data, we present a novel Chinese-based dual-channel model, DSI-BTCNN, which leverages deep learning to discern patterns indicative of suicidal ideation. Our model is designed to process Chinese data and capture the nuances of text locality, context, and logical structure through a fine-grained text enhancement approach. It features a complex parallel architecture with multiple convolution kernels, operating on two distinct task channels to mine relevant features. We propose an information gain-based IDFN fusion mechanism. This approach efficiently allocates computational resources to the key features associated with suicide by assessing the change in entropy before and after feature partitioning. Evaluations on a customized dataset reveal that our method achieves an accuracy of 89.64%, a precision of 92.84%, an F1-score of 89.24%, and an AUC of 96.50%, surpassing TextCNN and BiLSTM models by an average of 4.66%, 12.85%, 3.08%, and 1.66%, respectively. Notably, our proposed model has an entropy value of 81.75, which represents a 17.53% increase compared to the original DSI-BTCNN model, indicating a more robust detection capability. This enhanced detection capability is vital for real-time social media monitoring, offering a promising tool for early intervention and potentially life-saving support.

## 1. Introduction

Suicide is a critical global health issue, ranking as a leading cause of death with over 700,000 fatalities annually [1,2]. Suicidal ideation, intricately linked to mental health, is particularly susceptible to the exacerbating effects of conditions like depression, anxiety, and PTSD [3]. Consequently, accurately identifying suicidal ideation and classifying those at risk are imperative. The early detection of suicidal ideation is acknowledged as the most effective strategies for suicide prevention [4]. Given the scarcity of medical resources, achieving real-time, comprehensive detection and intervention poses a significant challenge. Thus, an urgent need exists for an autonomous, economical, and scalable method to detect suicidal ideation in real time for widespread populations.

The evolving emotional discourse on social media has opened new avenues for suicidal ideation detection [4,5]. Previous studies have highlighted the effectiveness of AI, particularly in the fields of NLP and machine learning, in automatically identifying suicidal expressions on social media [6,7,8,9]. In China, the Sina Weibo platform (Weibo) has been a focal point for research [10,11]. Yet, the brevity, sparse vocabulary, and irregular linguistics of microblogs intensify the challenge of feature sparsity. This necessitates the deployment of detection models endowed with sophisticated feature extraction capabilities to effectively navigate the complexities of such data.

Progress in this field faces several challenges. Initially, the unstructured nature of microblog data poses a significant barrier, complicating the extraction of valuable information. Despite the partial alleviation of feature sparsity offered by CNNs and their advanced versions through proficient local feature extraction [12,13], their intrinsic shortcomings are still evident. The monolithic architecture of CNN, dependent on layer deepening for performance gains, can lead to extended training times and diminished global contextual understanding. In response, this study proposes a paradigm shift towards a parallel approach to alleviate the strain imposed by layer deepening model architectures. By employing convolution kernels of varying sizes, this study aims to broaden the scope of the CNN model’s text feature extraction capabilities, thereby capturing a richer tapestry of local features across various levels of fine granularity. More importantly, we recognize the varying significance of features from two channels under different circumstances. To enhance the model’s focus on critical suicide indicators, we introduce an information gain mechanism during the feature fusion process.

Furthermore, sentence-level classification for detecting suicidal intent is complex, requiring an understanding of context, textual order, and expression nuances. While CNN is adept at capturing local features, BiLSTM provides a bidirectional perspective, allowing for comprehensive contextual understanding. However, the integration of these approaches in hybrid models for feature extraction presents its own challenges [14,15]. The fusion of these models sometimes results in reduced performance, where the combined model underperforms its individual components. This highlights the need for an innovative approach: partitioning models into specialized feature extraction channels, then intelligently merging them at the output layer to amplify overall efficacy.

Moreover, there is a pronounced scarcity of authoritative Chinese datasets in the domain of suicidal ideation detection, with the majority being dominated by English textual data. Such cultural disparities can impede the efficacy of these datasets when transposed to other platforms, notably China’s Weibo. Weibo’s diverse virtual communities, including the Weibo Tree Hole and depression-related Super Topics, are replete with comments indicative of suicidal ideation, making it an essential platform for our detection task.

To tackle the identified challenges, this study introduces a Chinese dataset developed from “Super Topic” on Weibo and proposes a novel model named dual-channel automatic detection model of suicidal ideation based on BiLSTM and TextCNN with parallel multi-kernel (DSI-BTCNN). Our model harnesses the strengths of TextCNN in local feature extraction and BiLSTM in sequence context modeling, adeptly adapting to the nuances of microblog content.

The contributions of this paper are multifaceted:

- We introduce a novel Chinese dataset derived from Weibo, filling a significant gap in the research area of Chinese-centric data;
- Our proposed DSI-BTCNN model integrates the feature extraction capabilities of two distinct deep learning networks within a dual-channel framework, significantly improving the model’s accuracy in detecting suicidal ideation;
- The study emphasizes the benefits of a parallel multi-kernel architecture, deploying diverse kernel sizes to refine the model’s feature representation capabilities;
- We innovate by incorporating an information gain-based dynamic feature attention fusion network, dynamically modulating the fusion of diverse features. Our experiments demonstrate notable enhancements over baseline models.

This manuscript is structured as follows: Section 2 reviews the relevant literature on suicide risk detection via social media. Section 3 describes the DSI-BTCNN model. Section 4 details the creation of the dataset. Section 5 presents the experimental setup, including the baseline models and evaluation metrics. Section 6 provides a thorough analysis of the results. Section 7 discusses the implications of these findings. The paper concludes in Section 8 with a summary, limitations, and future research.

## 2. Related Works

On social media platforms, suicide ideation detection mainly uses machine learning technology to analyze the content of users’ social posts, including suicide text classification, suicide information inference, or suicide risk group identification. In the following, a systematic literature review is conducted from the perspective of CNN, RNN, and their combination models, respectively.

### 2.1. CNN-Based Detection of Suicidal Ideation

CNN is pivotal in detecting suicidal ideation on social media due to its robust feature extraction capabilities. Orabi et al. [12] compared CNN and RNN for identifying individuals at risk of suicide on Twitter, with CNN demonstrating superior performance. Allen et al. [16] enhanced CNN with linguistic queries and word count dictionaries, treating each post and user profile holistically, achieving a Macro F1-score of 0.5. Kim et al. [17] introduced the TextCNN model, pioneering a novel approach for text-based recognition of suicidal ideation. Building on Kim’s work, Yao et al. [13] developed a CNN-based framework tailored for detecting suicidal behavior among opioid users, achieving an impressive F1-score of 96% by balancing processing speed and accuracy. Addressing the common issue of information scarcity in CNN models, Li et al. [18] integrated Word2Vec embeddings with TextCNN, devising a multi-feature CNN model that correlates dictionary terms, user posting times, and social cues, achieving an accuracy of over 88% in identifying suicidal users and Chinese microblog content.

### 2.2. RNN-Based Detection of Suicidal Ideation

The local feature extraction in CNNs may fail to capture long-range dependencies crucial for classifying certain suicide tendencies, potentially impairing performance. In contrast, RNN leverages their recurrent mechanisms to process text by integrating the current input with the previous hidden state, thus capturing contextual relationships within the data. In practical applications, Alabdulkreem et al. [19] employed RNNs to analyze tweets from Arab women, discerning depression and associated suicide risks. Matero et al. [20] introduced a dual RNN model to parse suicide-related discussions on Reddit, aiming to identify at-risk individuals. However, their approach faced limitations in handling diverse contextual information, necessitating further enhancement of the model’s performance.

Addressing the pervasive issue of gradient vanishing in standard RNN, which impedes model efficacy, advanced variants such as the long short-term memory (LSTM) and bidirectional long short-term memory (BiLSTM) networks have been developed. Research by Kancharapu [21] and Deepa J et al. [22] has substantiated the efficacy of LSTM in the detection and prediction of suicide-related tweets. Almars [23] introduced an Arabic depression text classification model that integrates BiLSTM with an attention mechanism, achieving a 3% improvement in accuracy over the standard BiLSTM model. Kancharapu et al. [24] further employed a model with three semantically distinct BiLSTM frameworks to forecast suicide rates during pandemics. Their findings reveal that the BiLSTM model outperforms CNN, RNN, LSTM, and other neural network architectures, boasting an accuracy of 86.47%. This superior performance is attributed to the BiLSTM’s enhanced capability to capture and represent the nuances of suicidal ideation.

### 2.3. CNN-RNN Hybrid-Based Detection of Suicidal Ideation

As deep learning advances, hybrid models that amalgamate CNN and RNN have emerged as a focal point in research. Sawhney et al. [25] introduced a CNN-LSTM hybrid, demonstrating its superior accuracy of 81% over standalone models in detecting suicidal ideation in text, thereby validating the efficacy of integrated approaches. Kour [15] and Tadesse et al. [26] furthered this research by constructing the CNN-BiLSTM and LSTM-CNN models, respectively. These models achieved an accuracy of 94%, underscoring the enhanced detection capabilities afforded by hybrid architectures. Despite these strides, the capacity for feature extraction within single-layer CNN and RNN frameworks remain constrained. To address this, Priyamvada et al. [27] employed a stacked CNN with a two-layer LSTM model in a hybrid model for suicide risk assessment. This approach yielded a nearly 5% improvement in recognition accuracy over models relying solely on CNN, highlighting the potential of deeper integration for more nuanced detection.

To address the limitations in capturing long-term dependencies and local context, recent studies have incorporated attention mechanisms into hybrid models. Renjith et al. [28] introduced an attention layer prior to the convolutional layer, enabling the model to more effectively extract subtle features from suicide-related texts, with reported accuracy and F1-scores exceeding 90%. Chadha et al. [29] proposed the ACL model, which employs attention to hone in on critical data details and specific lexical items, thereby facilitating a more nuanced understanding of potential suicide cues within textual content. However, this model primarily targets linguistic and semantic features. In response, Zogan et al. [30] developed the MDHAN model, utilizing a dual-level attention mechanism to encode tweets and assess the significance of each tweet and word. This approach bolsters the model’s interpretability and, even in cases where textual features are subtle, achieves a detection accuracy of 89.5%.

Table 1 summarizes a brief comparison of the described methods.

## 3. Methodology

### 3.1. Model Detection Pprocedure

**Figure 1:**
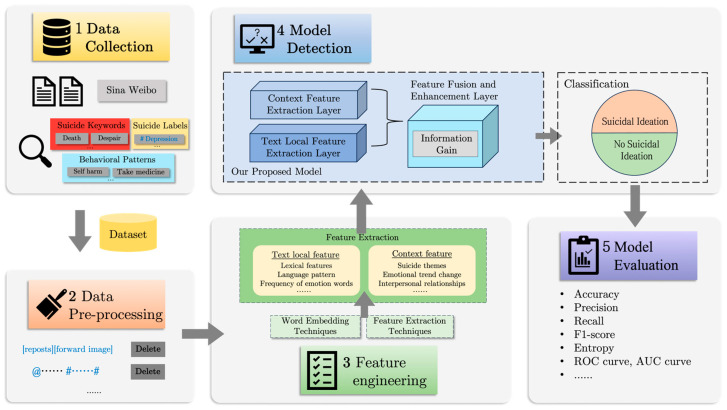
An overview of our model detection mechanism.

This study introduces a five-stage framework for text classification, using machine learning techniques to meticulously analyze the content of a user’s microblogs. The framework is depicted in Figure 1 and will be detailed in the following sections. Our model, tailored for deployment on Weibo, is adept at detecting suicidal ideation by classifying relevant text and informing the execution of pertinent intervention strategies.

### 3.2. Model Architecture

**Figure 2:**
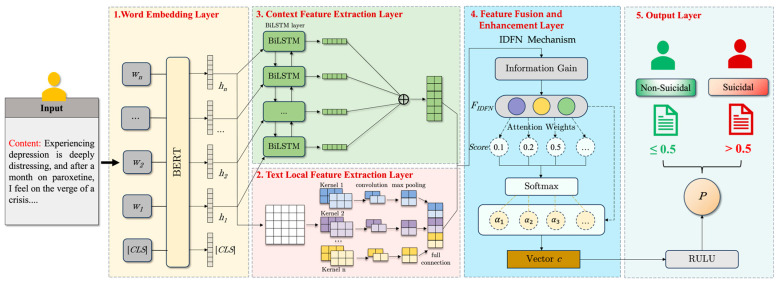
The architecture of the proposed model.

In this paper, we introduce the DSI-BTCNN model, a deep learning model designed for the detection of suicidal ideation. This model harnesses the local feature extraction prowess of TextCNN and the contextual feature capture capability of BiLSTM within a dual-channel architecture. The framework’s architecture is illustrated in Figure 2.

The model comprises the following components:

**(1)**  **Word Embedding Layer:** utilizing a pre-trained BERT model, this layer distills contextual features from the microblog corpus, generating word embeddings that enrich the input for subsequent layers;
**(2)**  **Text Local Feature Extraction Layer:** multiple parallel convolutional kernels of varying sizes operate to extract multi-scale local features, which are concatenated to form a refined feature set;
**(3)**  **Context Feature Extraction Layer:** comprising two LSTM layers, this component processes sequences in both directions, swiftly grasping sequence dependencies to extract contextual features;
**(4)**  **Feature Fusion and Enhancement Layer:** This stratum amalgamates features extracted from the preceding layers, employing a bespoke information gain-driven dynamic fusion mechanism to augment the model’s acuity in identifying pivotal elements within microblog texts. Consequently, this refines the articulation of the model’s global feature representation;
**(5)**  **Output Layer:** Fully connected layers further refine the global features. A final layer with an activation function is employed to produce the model’s output, namely, the probability of suicidal ideation classification, which informs the classification decision.

#### 3.2.1. Word Embedding Layer

Initially, the microblog text must be fed into the model’s word embedding layer, where it is transformed into a continuous vector represented by the BERT model, serving as the model’s input.

Let S represent a sequence of microblog text, comprising a succession of words:

(1) $$S_{s}=[w_{1},\;w_{2},\;\ldots ,\;w_{T}]$$

where $w_{t}$ denotes the t-th word, and T represents the sequence’s length. Each word is then indexed by $i_{t}$ within a pre-defined vocabulary V, curated based on word frequency in the training corpus and encompassing the top N most frequent words.

Subsequently, the embedding matrix *E* is employed to ascertain the word vector aligned with each index $i_{t}$. We integrate the methodologies of token embeddings, segment embeddings, and positional embeddings. The amalgamated embedding matrix is articulated as

(2) $$E=E_{t}+E_{s}+E_{p}$$

Here, $E_{t}$ corresponds to the word embeddings derived from token embeddings, reflecting the semantic import of individual words. $E_{s}$ denotes the embeddings attributed to segment embeddings, encapsulating the contextual essence of sentences or paragraphs. Given the absence of recursive or convolutional structures in the Transformer architecture, positional embeddings are indispensable, yielding the matrix $E_{p}$ that imparts positional information within the sequence.

For each word $w_{i}$ in the sequence, it undergoes self-attention and multi-head attention mechanisms to produce a contextually embedded vector $h_{i}$, which is formulated as

(3) $$h_{i}=\mathrm{BERT}\;(w_{i},E,\Theta )$$

In this equation, Θ encapsulates the BERT model’s parameters, comprising the self-attention layer’s weights and the deep feedforward network’s coefficients.

The word embedding layer’s output is a sequence of contextually enriched embedding vectors $H[h_{1},h_{2},\ldots ,h_{T}]$, which serves as the subsequent layer’s input. Subsequently, we establish dual channels: one dedicated to the Text Local Feature Extraction Layer and the other to the Context Feature Extraction Layer.

#### 3.2.2. Text Local Feature Extraction Layer

The role of this layer in the TextCNN model involves deploying multiple convolution kernels of varying scales to meticulously sample local features across different spatial extents in parallel. The extraction of local features is executed by a series of convolution kernels of distinct sizes that concurrently traverse the input sequence, capturing local contextual information within varying window dimensions.

Let K denote the total number of convolutional kernels, where $k_{i}$ signifies the dimensions of the i-th kernel. Let $C_{i}$ represent the output feature map from the i-th convolutional layer. For each kernel $k_{i}$ and time step t, the convolution operation is articulated by the following formula:

(4) $$C_{i,t}=Conv_{i}(h_{t})=\sum_{j=0}^{k_{i}-1}h_{t-j}w_{i,j}$$

where $w_{i,j}$ corresponds to the weight of the j-th position in kernel i, and $C_{i,t}$ is the feature map generated at time step t.

Furthermore, the rectified linear unit (ReLU) activation function is employed to incorporate non-linearity, thereby enriching the model’s expressive capacity:

(5) $$a_{i,t}=\mathrm{ReLU}(C_{i,t})$$

Here, $a_{i,t}$ is the activated feature after the ReLU transformation.

Subsequently, to distill the most salient local features and curtail the parameter count, we implement a max-pooling operation, yielding novel feature representations as delineated:

(6) $$p_{i}=\mathrm{MaxPooling}(a_{i,1},\;a_{i,2},\ldots ,\;a_{i,T-k_{i}+1})$$

Ultimately, the outcomes of max-pooling from the convolutional kernels across various channels are concatenated to forge an extensive feature vector, as expressed in Equation (7):

(7) $$x=p_{1}\;⊕p_{2}\;⊕p_{3}\ldots ⊕p_{n}$$

where ⊕ symbolizes the concatenation operation.

This approach facilitates a multi-core parallel processing mode, enabling the CNN to function as a versatile sampler across diverse window sizes, thereby acquiring feature representations of varied granularities. Consequently, the clarity of the local feature maps is enhanced.

#### 3.2.3. Context Feature Extraction Layer

In the complementary channel, we employ a BiLSTM model, comprising two LSTM layers that sequentially process the microblog text in both forward and reverse directions. This architecture adeptly captures the contextual nuances and logical fabric of the text, effectively extracting potential indicators of suicidal ideation. The suitability of this approach for detecting suicidal inclinations in microblog texts is evident.

Each LSTM unit comprises four components: the input gate $i_{t}$, the forget gate $f_{t}$, the cell state $C_{t}$, and the output gate $o_{t}$. These components govern the influx and modification of information, as delineated in Equations (8)–(15):

(8) $$i_{t}=\sigma (W_{i}\left[h_{t-1},x_{t}\right]+b_{i})$$

(9) $$f_{t}=\sigma \left(W_{f}\left[h_{t-1},x_{t}\right]+b_{f}\right)$$

(10) $$C_{t}=\tanh\left(W_{C}\left[h_{t-1},x_{t}\right]+b_{C}\right)$$

(11) $$C_{t-1}=f_{t}\times C_{t-1}+i_{t}\times C_{t}$$

(12) $$\vec{h_{t}}=o_{t}\times tanh(C_{t})$$

(13) $$\overset{\leftarrow }{h_{t}}=o_{t}\times tanh(C_{t})$$

(14) $$o_{t}=\sigma (W_{o}\left[h_{t-1},x_{t}\right]+b_{o})$$

(15) $$\tanh\left(x\right)=\frac{e^{x}-e^{-x}}{e^{x}+e^{-x}}$$

Here, sigmoid denotes the sigmoid function, while tanh represents the hyperbolic tangent function. W and b correspond to the weight matrix and bias vector, respectively.

The culminating layer output merges the forward and backward hidden states at each timestep t, yielding a comprehensive context representation:

(16) $$H_{t}^{BiLSTM}=\left(\vec{h_{t}}:\overset{\leftarrow }{h_{t}}\right)$$

where $H_{t}^{BiLSTM}$ encapsulates bidirectional contextual insights, furnishing pivotal contextual cues for subsequent detection tasks.

#### 3.2.4. Feature Fusion and Enhancement Layer

In this stratum, transcending conventional feature concatenation techniques, we incorporate an information gain-driven dynamic feature attention fusion network (IDFN). This network’s information gain and attention mechanism dynamically modulate the fusion weights of features extracted from the preceding layers, allowing the model to discern and learn the significance of each feature in the detection of suicidal ideation.

Initially, the features x and $H_{i}^{BiLSTM}$, derived from the Text Local Feature Extraction Layer and the Context Feature Extraction Layer, respectively, undergo weighting based on information gain. We introduce the formulas for calculating information gain as follows:

(17) IG(x)=H(x)−H(x|y)

(18) $$w_{x_{i}}=\frac{IG(x_{i})}{\sum_{i}IG(x_{i})}$$

(19) $$w_{h_{t}}=\frac{IG(h_{t})}{\sum_{t}IG(h_{t})}$$

where H(x) is the entropy of the feature, and H(x|y) is the conditional entropy of x given a specific label.

Subsequently, we amalgamate the two weighted feature sets to derive the composite feature representation, as delineated in Equation (20):

(20) $$F_{IDFN}=\sum_{i}w_{x_{i}}⊙x_{i}+\sum_{t}w_{h_{t}}⊙h_{t}$$

where ⊙ denotes element-wise multiplication. The resultant feature representation $F_{IDFN}$ encapsulates the synergistic benefits of both local and contextual features.

To augment these features and distill salient information, we implement an adaptive attention mechanism that apportions differential weights to each feature element. This mechanism leverages a learnable query vector q, which interacts with the feature representation to generate an attention score for each feature, and it is expressed as follows:

(21) $${score}_{t}=q^{T}\tanh\left(WF_{IDFN}+b\right)$$

Continuing with the process, we then proceed to normalize the attention scores using the softmax function, which ensures that the sum of the attention weights across all timesteps equals one:

(22) $$\alpha_{t}=\frac{\exp\left({score}_{t}\right)}{\sum_{t^{\prime }=1}^{T}\exp\left({score}_{t^{\prime }}\right)}$$

In this equation, $\alpha_{t}$ represents the normalized attention weight, indicating the significance of the feature at each timestep t in the final output.

Subsequently, these attention weights are utilized to compute a weighted sum of the features, yielding the augmented global feature vector c:

(23) $$c=\sum_{t=1}^{T}\alpha_{t}F_{IDFN}$$

This refined fusion mechanism ensures the model’s agility in assigning relevance to features, thereby enhancing the efficacy of suicidal ideation detection.

#### 3.2.5. Output Layer

The output layer’s objective is to ascertain the probabilistic classification of suicidal ideation, indicating the likelihood that the input text exhibits suicidal inclinations. Initially, the augmented feature vector c is input into a series of fully connected layers designed to map the features onto latent scores for the classification task, employing the ReLU activation function to enrich the decision boundary’s complexity:

(24) $$z=\sigma \left(W_{fc}c+b_{fc}\right)$$

(25) $$z^{\prime }=\mathrm{ReLU}(z)$$

Subsequently, the output of these layers is condensed to a single node, with the resultant value being transformed into a probability via the sigmoid function, signifying the likelihood of the microblog text containing suicidal ideation given feature vector c:

(26) $$P(y=1|c)=\sigma (z^{\prime })$$

A value exceeding 0.5 prompts the classifier to deem the text as indicative of suicidal ideation; otherwise, it is categorized as non-suicidal.

## 4. Datasets

The integration of state-of-the-art machine learning in the detection of suicide ideation invariably engenders concerns regarding privacy and ethics. Securing extensive, high-fidelity, and significant data while safeguarding user confidentiality presents a formidable challenge. This predicament is exacerbated by an absence of specialized public datasets tailored to Chinese user demographics. To surmount these obstacles, we crafted a novel dataset derived from Weibo that was meticulously curated and preprocessed to furnish robust data support for our investigation.

### 4.1. Data Collection and Annotation

Weibo features a “Super Topic” functionality where users can contribute their comments. Our objective is to source data from Super Topics on Weibo. The melancholy related Super Topics encompass those focused on depression, such as “Depression Super Topic” and “Depression Patients Super Topic”, which aggregate a substantial user base expressing pronounced suicidal tendencies in their posts, utilizing high-risk terminology like “wrist cutting”, “charcoal burning”, and “hanging”. In contrast, unrelated depression Super Topics, such as those centered on daily life segments like VLOGs, pets, and giant pandas, facilitate the sharing of everyday experiences devoid of explicit suicidal indicators. To ensure dataset equilibrium, our study employs a systematic web-crawling approach to collect 40,000 Super Topic data entries from both depression-related and unrelated Super Topics, representing a spectrum of suicidal expression from overt to subtle.

To safeguard the precision of user annotations, we rely on the Chinese suicide dictionary [31] and strictly followed the annotation criteria delineated by Meng [32]. To refine our annotation process, we enlist four psychology experts for independent reviews, resolving discrepancies through consensus to ensure definitive conclusions.

Table 2 illustrates select instances of microblog entries, both indicative and non-indicative of suicidal ideation.

### 4.2. Data Pre-Processing

The linguistic irregularity inherent in microblog expressions, replete with platform-specific characters, can introduce noise detrimental to the suicide detection model’s performance, thereby impacting the analysis of suicidal ideation. To mitigate this, rigorous data preprocessing is imperative to ensure data integrity and utility. Our preprocessing steps include the following:

- Eliminating HTML tags, URLs, special characters, and emojis to mitigate noise;
- Trimming extraneous whitespace and punctuation and normalizing numerical data;
- Conducting word segmentation to transform text into discrete words or phrases for computational processing;
- Excising microblog-specific characters, such as “Super Topic Influencer”, which are nonsensical in sentiment analysis;
- Discarding microblog entries shorter than five characters, as they convey minimal emotional content post-processing;
- Stripping hashtags that denote microblog topics, e.g., #MyFavoriteHongKongTVSeriesinTVB#;
- Removing @user mentions, as they are irrelevant to the sentiment analysis, such as @Maston;
- Eradicating microblog behavior-related characters, like ‘[reposts]’, which appear in shared content.

After these meticulous preprocessing steps, we culled a dataset comprising about 80,000 microblogs. In this study, we employ desensitization techniques to anonymize personal information, assigning unique identifiers to protect user privacy. Our data are published at https://github.com/Zoeeyue/data, accessed on 21 January 2025. Refer to the file named “datasets” in the data repository.

## 5. Experiments

### 5.1. Experimental Setup

In our research, to ascertain the uniformity and veracity of the experimental outcomes, we meticulously assembled a Chinese dataset as detailed in Section 4. The dataset is divided into two sets: a training set and a test set. A total of 80% of the data are used for model training, while the remaining 20% are used to evaluate model performance. This partitioning methodology was applied consistently across all experiments to uphold the uniformity of the experimental milieu.

Our experimental procedures were conducted within a Python environment, utilizing PyTorch 2.0, a sophisticated deep learning framework. During model training, we employed the Adam optimizer to bolster training efficacy, augment the model’s adaptability to novel data, and effectively mitigate overfitting. Furthermore, we selected the cross-entropy loss function as the model’s loss criterion to precisely quantify the divergence between the model’s predictions and the actual labels.

### 5.2. Baseline

To benchmark our model’s efficacy, we selected a spectrum of established machine learning and deep learning algorithms, categorized into machine learning and deep learning groups.

#### 5.2.1. Machine Learning Group

- Support Vector Machine (SVM): we utilize an SVM with an RBF kernel for microblog feature processing, setting parameter C to 0.1 and γ as the inverse of the feature space’s dimensionality;
- Naive Bayes (NB): a multinomial Naive Bayes classifier is utilized, calibrated with a smoothing parameter α of 0.001 to harmonize the smoothing effect with the data’s sparsity within the probabilistic framework;
- Random Forest (RF): constructed with an ensemble of 50 decision trees, and each tree is cultivated via bootstrap sampling.

#### 5.2.2. Deep Learning Group

Our deep learning group is bifurcated into CNN and RNN subgroups, showcasing the models’ capabilities in extracting local and contextual textual features, respectively.

**(1)**  **CNN Subgroup:**
  - CNN: utilizes varied convolution kernels and max-pooling, with a 100D input vector, ReLU, and Adam for efficient training;
  - TextCNN: Capitalizing on the local feature extraction of CNNs, this model utilizes multiple kernel sizes to process text data. We have set 128 convolution kernels across three distinct window sizes, maintaining a 100-dimensional input vector;
  - TextRCNN: a hybrid model that amalgamates the strengths of CNNs and RNNs, featuring a three-layer RNN structure with a 100-dimensional input vector.
**(2)**  **RNN Subgroup:**
  - RNN: our RNN model is structured with three layers, each with 100-dimensional input vectors, and employs an SGD-optimized learning rate of 0.001;
  - TextRNN: engineered to adeptly manage variable-length text sequences, this model mirrors the RNN structure, also with 100-dimensional input vectors;
  - LSTM: equipped with 100-dimensional input vectors, this model allocates 128 LSTM units per layer to strike a balance between complexity and computational efficiency;
  - BiLSTM: Enhancing the LSTM with a bidirectional propagation pathway, this model considers both antecedent and subsequent textual information. We configured 128 LSTM units for both the forward and backward components of the BiLSTM, with a 100-dimensional input vector.

### 5.3. Evaluation Metrics

To thoroughly assess the DSI-BTCNN model’s efficacy against baseline models, we employ a comprehensive suite of metrics: accuracy (Acc), precision (P), recall (R), and F1-score (F1). These metrics are precisely calculated from the confusion matrix elements using the following defined formulas:

(27) $$Acc=\frac{TP+TN}{TP+TN+FP+FN}$$

(28) $$R=TPR=\frac{TP}{TP+FN}$$

(29) $$P=\frac{TP}{TP+FP}$$

(30) $$F1=\frac{2\cdot P\cdot R}{P+R}$$

Additionally, we incorporate the ROC curve and its AUC as standard metrics for predictive accuracy, employing the following formulas:

(31) $$Specificity=\frac{TN}{TN+FP}$$

(32) FPR=1−Specificity

(33) $$AUC=\frac{1+TPR-FPR}{2}$$

Since our model employs information gain, in order to verify the validity of the model, we use entropy as a measure to assess the predictive uncertainty of the model:

(34) $$Entropy=1-\frac{1}{N}\sum_{i=1}^{N}H(p_{i})$$

After adjusting the entropy values to a normalized scale, we apply a transformation that maps them to the range of 0 to 1. In this context, a lower entropy value, which corresponds to a higher normalized score, signifies a model with greater confidence in its predictions, suggesting better performance.

## 6. Results

In this section, we evaluate the performance of machine learning and deep learning models on a Chinese suicidal ideation dataset, focusing on Word2Vec, GloVe, and BERT embeddings. Table 3 highlights BERT’s outstanding performance, significantly improving accuracy, F1-score, and AUC due to its advanced contextual understanding, which is crucial for interpreting the complex and ambiguous language of microblogs. Our findings reveal that deep learning models outperform traditional machine learning approaches. The TextCNN model, enhanced with BERT embeddings, leads in the CNN category with a 3% boost in accuracy, recall, F1-score, and AUC, showcasing its strong local feature extraction. In the RNN category, BiLSTM with BERT integration achieves an 84.68% accuracy rate, benefiting from its bidirectional context modeling.

We conducted an in-depth analysis to discern the impact of model architectures on performance. We sequentially integrated TextCNN with BiLSTM and vice versa, and the comparative outcomes are presented in Rows 3 and 4 of Table 4 to assess their synergistic effectiveness. The serial-amalgamated model outperformed its singular counterparts, with enhancements in accuracy, precision, and F1-score. Notably, the BERT-BiLSTM-TextCNN model achieved a 1% improvement in accuracy, a gain that can be attributed to the synergistic benefits of the combined models.

Further analysis, presented in Line 5 of Table 4, reveals that our DSI-BTCNN dual-channel model surpasses all previous models. With accuracy, precision, F1-score, and AUC peaking at 86.7%, 82.43%, 87.56%, and 95.64%, respectively; the model substantiates the efficacy of our parallel dual-channel design. This architecture enables each channel to independently undertake distinct feature extraction tasks, thereby bolstering the model’s performance.

**Figure 3:**
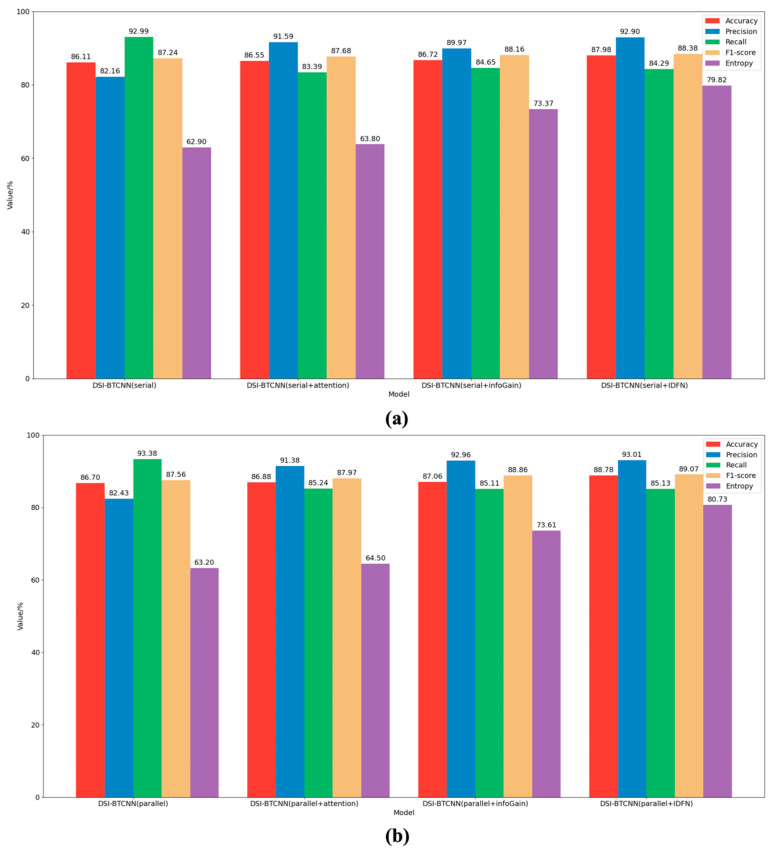
Model classification results with the introduced IDFN mechanism. (**a**) The performance of the IDFN mechanism in serial mode group. The DSI-BTCNN (Serial) model utilizes the BERT-TextCNN-BiLSTM architecture as delineated in Table 4; (**b**) The performance of the IDFN mechanism in parallel mode group.

Building upon the DSI-BTCNN model, we conducted an in-depth evaluation of the IDFN fusion mechanism’s utility. The results depicted in Figure 3 demonstrate significant performance improvements across both serial and parallel models upon the incorporation of the IDFN strategy. The parallel models consistently outperformed their serial counterparts in most key metrics, highlighting the superiority of dual-channel parallel feature processing capabilities. Specifically, the DSI-BTCNN(serial + IDFN) model achieved an accuracy of 87.98%, marking a 1.89% enhancement over the baseline serial model. The parallel model, DSI-BTCNN(parallel + IDFN), also attained an accuracy of 88.78%, which is nearly 1% higher than the serial model.

It is worth noting that the introduction of the IDFN strategy increased the entropy of both models by 16.92% and 17.53%, respectively. This suggests that the model has become more effective in focusing on key suicidal features and further confirms the role of IDFN in enhancing the model’s predictive stability.

Further ablation studies revealed the individual contributions of information gain and attention mechanisms to the fusion strategy. The findings indicate that the inclusion of both mechanisms enhances model performance, with information gain showing a slightly more pronounced improvement. Notably, when information gain was introduced, its entropy was over 10 times higher than that of the attention mechanism, indicating a superior ability in feature selection and focus. The combination of these two mechanisms within the IDFN led to optimal model performance, suggesting a synergistic effect. Our proposed model, DSI-BTCNN(parallel + IDFN), excelled as shown in Figure 3b, with an accuracy of 88.78%, a precision of 93.01%, and an F1-score of 89.07%. Compared to the original parallel model, these figures represent increases of 2.08%, 10.58%, and 1.51%, respectively. These results not only substantiate the positive impact of IDFN on model classification but also illustrate the complementary nature of information gain and attention mechanisms.

**Figure 4:**
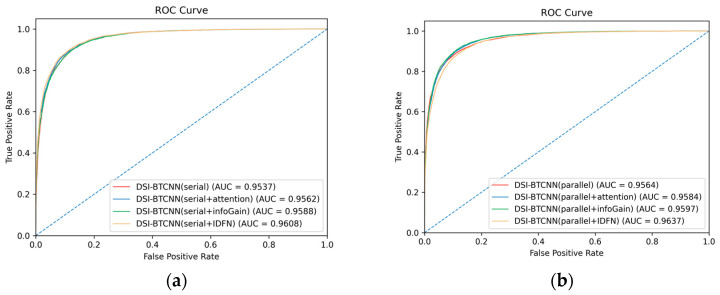
IDFN integration impact on AUC-ROC performance. (**a**) AUC-ROC performance of the IDFN mechanism in the serial model group; (**b**) AUC-ROC performance of the IDFN mechanism in the parallel model group.

To gain further insights into model performance, we compared the models in Figure 3 using AUC-ROC curves, as shown in Figure 4. The AUC values for various classifiers, presented in the legends, indicate that the combination of different models with our recommended fusion strategy yields results above 95%. Particularly, our proposed DSI-BTCNN(parallel + IDFN) model stands out with an impressive AUC of 96.37%, surpassing the original DSI-BTCNN model by 0.73%, demonstrating its exceptional ability to distinguish between the two target categories.

**Figure 5:**
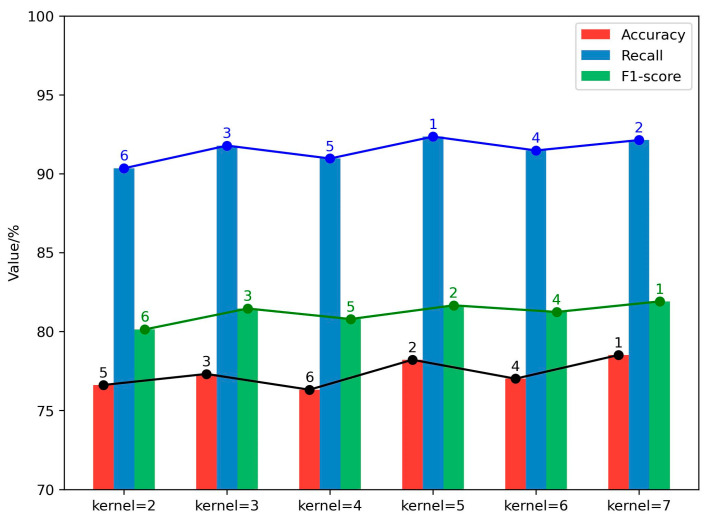
Comparative analysis of model performance across diverse kernel sizes. The number of vertices represents the order of the numerical value of each group.

We further investigate the influence of employing multiple convolution kernels in classifying suicide-related texts. Initially, we examined the impact of varying the size of convolution kernels. Our empirical findings suggest that, in contrast to utilizing a single large kernel, leveraging multiple smaller kernels can decrease the parameter count and computational load while retaining the necessary connectivity. This approach also enhances the model’s recognition capabilities. Experiments were conducted with kernel sizes ranging from two to seven, with the results depicted in Figure 5. The model demonstrated peak accuracy, recall, and F1-score for kernel sizes of five, seven, and three, successively followed by six, four, and two. Hence, this paper sequentially prioritizes kernel sizes as (5, 7, 3, 6, 4, 2).

After kernel size selection, we examine the influence of varying the quantity of convolutional kernels within the model. As illustrated in Table 5, there is an initial enhancement in feature extraction efficacy with an increase in the number of kernels, culminating in optimal model performance at a count of three kernels. Curiously, beyond this threshold, the model exhibits signs of overfitting. Notably, the DSI-BTCNN model, now with triple kernel capability, achieved a new benchmark in suicide ideation detection, with metrics reaching their peak with an 89.64% accuracy, an 85.90% precision, an 89.24% F1-score, and an AUC of 96.50%. When compared with the original proposed model (Table 4, Line 5), the enhancements are substantial, with increases of 2.94% in accuracy, 10.41% in precision, 1.68% in F1-score, 0.86% in AUC, and 1.06% in entropy, respectively. Compared to the average performance of the original TextCNN and BiLSTM models (Table 4, Lines 1 and 2), our model has seen a significant upswing in performance metrics, with accuracy rising by 4.66%, precision by 12.85%, the F1-score by 3.08%, and AUC by 1.66%. Consequently, we have designated this configuration as our final selection for the DSI-BTCNN model.

**Figure 6:**
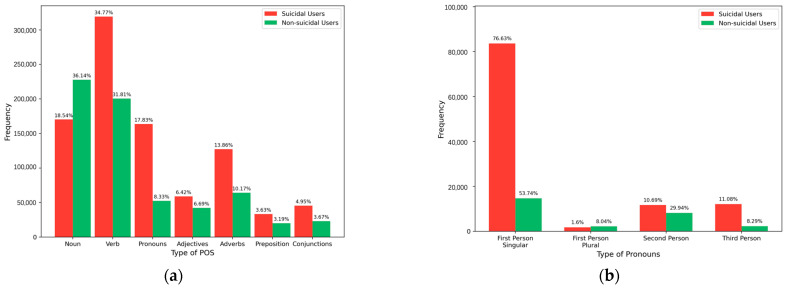
The proportion of POS in the suicidal and non-suicidal groups. (**a**) POS distribution in suicidal and non-suicidal users; (**b**) Pronoun distribution in suicidal and non-suicidal users.

To further explore the relationship between our model and linguistic features, we statistically analyze the POS distribution of different users. The results are shown in Figure 6a. Among suicidal users, the frequency of verb usage is the highest, accounting for 34.77%. Nouns follow, with a proportion of 18.54%. In contrast, the situation is reversed for non-suicidal users. This indicates that suicidal users tend to express themselves more through verbs, while non-suicidal users have a greater preference for nouns. Interestingly, we also conducted a statistical analysis of pronoun types. As shown in Figure 6b, the usage frequency of first-person singular pronouns among suicidal users is as high as 76.63%, which is significantly higher than 53.74% among non-suicidal users. This may imply that suicidal users are more self-focused, and their degree of self-focus is significantly higher than that of non-suicidal users.

**Figure 7:**
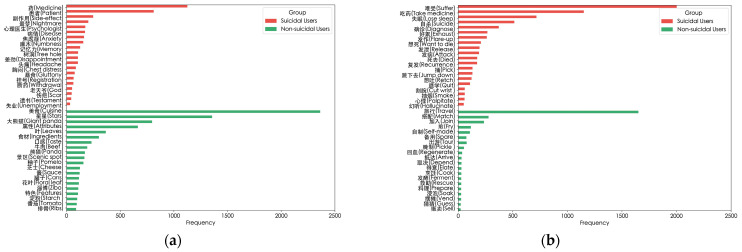

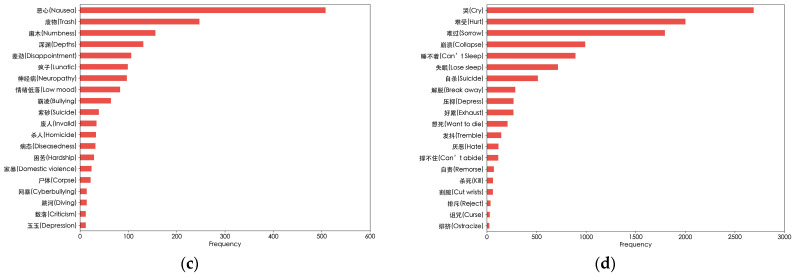
High-frequency words used by users in different groups. (**a**) High-frequency nouns used by suicidal and non-suicidal users; (**b**) High-frequency verbs used by suicidal and non-suicidal users; (**c**) High-frequency nouns with emotional characteristics used by suicidal users; (**d**) High-frequency verbs with emotional characteristics used by suicidal users.

To gain an in-depth understanding of the model’s ability to extract local text features, as shown in Figure 7, we present the top 20 verbs and nouns in each group, sorted by their log-likelihood ratio. As shown in Figure 7a,b, among suicidal users, high-frequency nouns such as “Medicine” and “Psychologist” and high-frequency verbs such as “Take Medicine” and “Diagnose” may imply that the users are likely suffering from mental illnesses or seeking medical intervention. This further reflects the potential association that may exist between these words and suicidal tendencies.

We also explore the impact of users’ emotional states on suicidal ideation. Referring to the Chinese suicide dictionary [31], we count the high-frequency verbs and nouns with semantic features among suicidal users. The results are presented in Figure 7c,d. Words like “Nausea”, “Disappointment”, “Cry”, and “Collapse” may suggest that users are experiencing the influence of severe negative emotions. These words complement the previously mentioned high-frequency words, jointly forming a unique language and semantic feature profile for suicidal users.

**Figure 8:**
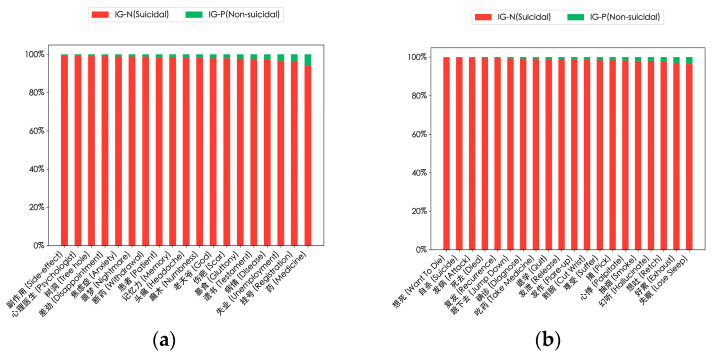

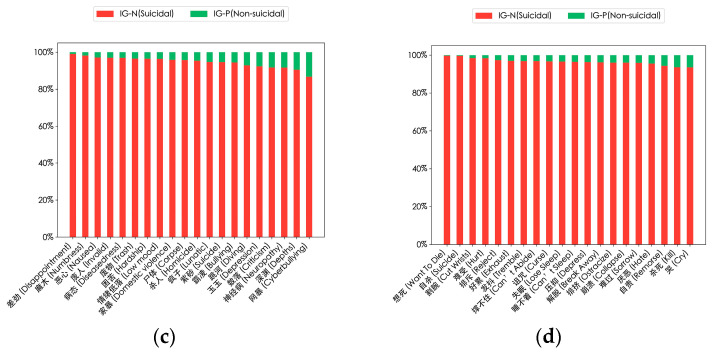
Results of the information gain of high-frequency words of suicidal users. Each bar corresponds to a word, representing the relative importance of its information gain value in suicidal users’ and non-suicidal users’ posts. (**a**) Information gain of high-frequency nouns; (**b**) information gain of high-frequency verbs; (**c**) information gain of high-frequency nouns with emotional characteristics; (**d**) information gain of high-frequency verbs with emotional characteristics.

In addition, to better distinguish between suicidal and non-suicidal users, we calculated the information gain of the high-frequency words in Figure 7. The results are shown in Figure 8, indicating their importance in the suicidal context (red bars) and non-suicidal context (green bars). These words are key semantic vocabularies for the model to identify suicidal ideation.

We also carry out a case study to explore the consistency between the DSI-BTCNN model’s recognition effect and the above-mentioned conclusions. In Case 1, there are a small number of high-frequency words. After increasing the number of high-frequency nouns with high information gain in Case 2, the probability calculated by the model increases from 92.50% to 96.36%. This indicates that high-frequency nouns with high information gain, such as “Anxiety”, can strengthen the expression of suicidal tendencies. In Case 3, with the addition of semantic high-frequency nouns like “Invalid”, “Lunatic”, and “Trash”, the probability reaches 99.87%, an increase of 3.51% compared to Case 2, demonstrating the effectiveness of semantic high-frequency nouns in conveying suicidal ideation.

Similarly, in Case 5, after adding high-frequency verbs such as “quit”, the probability increased by 3.24% compared to Case 4. In Case 6, after adding semantic high-frequency verbs, the probability even increased by 7.16%. This proves the crucial role of high-frequency words in our proposed model’s identification of suicidal ideation and the effectiveness of information gain in feature selection.

We also explore the relationship between the model and pronouns. By comparing Case 7 and Case 8, we find that the DSI-BTCNN model performs remarkably well in an environment with a higher density of first-person singular pronouns.

Finally, in Case 9, which contains multiple high-frequency words and semantic words, the probability of the model predicting suicidal ideation is as high as 99.98%. This fully validates the high consistency between our proposed model and these linguistic and emotional features, indicating that these features are highly effective in identifying suicidal tendencies.

## 7. Discussion

Preventing suicide remains a pressing global imperative, highlighting the necessity for proactive detection of suicidal ideation. Our investigation accentuates the pivotal role of social media in the early detection of suicidal tendencies, marking a departure from traditional identification methods. Our study advocates for an innovative model architecture that transcends standard approaches by incorporating a dual-channel mechanism, thereby enhancing the model’s capacity for text classification. This model, underpinned by a sophisticated parallel multi-kernel processing framework, adeptly navigates through a variety of textual features and stylistic nuances, ensuring the precise identification of individuals and groups at risk.

### 7.1. Effectiveness of the Dual-Channel Architecture

Our empirical studies confirm that the dual-channel architecture substantially elevates the model’s efficacy, as illustrated in Table 4. The results validate the structural superiority of our model and are consistent with previous studies [33,34,35].

The success of our model can be ascribed to several pivotal factors.

Firstly, the dual-channel configuration facilitates the extraction of nuanced features from microblog data. Social media texts, often replete with subtleties such as metaphors and complex linguistic constructs, pose a formidable challenge due to their cultural and stylistic diversity. However, our model’s complementary branches adeptly surmount these obstacles by harnessing the strengths of both BiLSTM and TextCNN. TextCNN adeptly seizes local textual elements, such as keywords and phrases, while BiLSTM excels at unraveling sequential patterns and contextual nuances, thereby augmenting the model’s interpretability.

Moreover, the dual-channel paradigm effectively circumvents the limitations inherent in serial model configurations. While serial models may incrementally enhance accuracy, they are prone to inter-layer dependencies that could result in the omission of critical features, as indicated in references [36,37]. The dual-channel strategy, by allowing for the independent design and optimization of each branch, alongside concurrent processing of varied data, ensures robust performance even when one channel encounters data-specific challenges.

### 7.2. Advantages of the IDFN Fusion Mechanism

Empirical evaluations have demonstrated significant enhancements in the model’s classification capabilities following the integration of our proposed IDFN mechanism, as depicted in Figure 3 and Figure 4. There are several compelling reasons for these improvements.

Firstly, diverging from conventional feature fusion approaches [38,39], our IDFN strategy embodies a theoretical framework for optimal feature selection that fortifies the feature selection process within a dual-channel architecture. For instance, one channel may concentrate on lexical features, such as the occurrence of specific keywords, while the other channel assesses the sentiment or emotional tone of the text. The IDFN strategy harmonizes these varied features, employing information gain to assign appropriate weights and leveraging the attention mechanism to focus on the most informative elements. This approach leads to a more precise detection of suicidal ideation. The combination of dual-channel processing and information gain-based weight allocation introduces innovative perspectives and methodologies for deep learning in the domain of text processing.

Secondly, compared to traditional feature concatenation or averaging techniques [40,41], our proposed fusion strategy employs information gain to allocate fusion weights, thereby ascertaining the significance of different features. This enables the model to adapt the fusion weights based on text length, contextual scenarios, and task requirements, mitigating the inherent information loss associated with feature concatenation. Furthermore, by integrating the attention mechanism, the IDFN mechanism refines the weight calibration of features, allowing the model to concentrate on those that have a more substantial impact on suicidal ideation within specific contexts. This is crucial for understanding the etiology of suicidal ideation and for the development of preventive measures.

Additionally, given that social media text constitutes sequences of variable lengths, the IDFN mechanism optimizes resource allocation. Information gain provides a basis for weight assignment to the attention mechanism, enabling the model to allocate processing resources more efficiently across texts of differing lengths. Consequently, the model can allocate greater resources to features that carry more information and disregard those with lower information gain. This enhances the model’s text mining capabilities, allowing it to discern users’ needs, interests, and emotions from their posts, thereby improving the overall computational efficiency in detecting suicidal ideation.

### 7.3. Efficacy of Parallel Multicore Architectures

We posit that the deployment of parallel multi-scale convolutional kernels can markedly enhance model performance, a hypothesis corroborated by the empirical evidence presented in Table 5.

The efficacy of this approach can be attributed to several compelling factors.

Initially, convolutional kernels of varying sizes enable the acquisition of distinct granular local features. Kernels of a smaller scale adeptly seize minute features, such as isolated words or phrases, while larger kernels are adept at capturing the relational nuances and interdependencies among words. This includes the synergistic effects of word combinations that may have pronounced features when conjoined but less distinct when isolated, as well as contexts demanding inferential reasoning. It is imperative to note that the quantity of convolutional kernels should be judiciously tailored to the demands of the specific data task. Our model functions akin to a magnifying glass, capable of dynamic scaling to broaden the analytical purview, amplify the extraction of key information across various scopes, and mitigate the model’s undue reliance on particular features.

Additionally, the parallel architecture significantly mitigates the performance bottlenecks associated with the traditional sequential stacking of CNN layers. Excessive layer stacking can impose a substantial computational load and precipitate overfitting issues, as referenced in [42]. The parallel configuration facilitates the extraction of a richer feature set within a more compact network, hastening convergence and diminishing the susceptibility to overfitting. This architecture permits the independent optimization of convolutional kernels of varying scales, allowing for customized training regimens that cater to the unique attributes of each kernel. Such an approach not only amplifies the model’s capacity for feature expression but also attenuates the incidence of feature sparsity.

### 7.4. Word Frequency, Sentiment Analysis and Case Study

In this study, by analyzing the usage frequencies of different POS types and specific words in the texts of suicidal users and non-suicidal users and calculating the information gain of key words, we deeply explore the importance and effectiveness of linguistic and semantic features in differentiating between the two types of users. This provides a basis for our model’s robustness. We obtained the following findings.

First, linguistic features play a guiding role in model improvement. As shown in Figure 6, the POS usage habits of different groups of people vary. Linguistic features are reflections of users’ inner states. In particular, the use of first-person verbs, nouns, and singular pronouns is considered to be related to suicidal ideation, which is consistent with previous research [43]. Moreover, as can be seen from the cases in Table 6, our model demonstrates excellent recognition efficiency in an environment rich in verbs, nouns, and pronouns. Therefore, in suicide-related discourse, it is necessary to further explore fine-grained language features, including examining different grammatical situations of pronouns according to reference [43]. Understanding the psychological impacts associated with these cases can provide deeper insights into the cognitive and emotional processes related to suicidal ideation.

Second, negative semantic words are important linguistic signals of suicide risk. In Figure 7, we focus on the high-frequency and semantic words used by suicidal users. Users with suicidal tendencies frequently use strongly negative semantic words such as “Nausea” and “Disappointment”, as well as specific semantic high-frequency nouns like “Invalid”. The accumulation and specific combinations of these words may imply the continuous intensification of negative emotions and severe as self-cognitive biases, thus promoting the emergence of suicidal ideation, which is consistent with the conclusions of previous studies [44,45]. As can be seen from Cases 6 and 9, when emotionally charged high-frequency words such as “Sorrow” and “Want to die” are combined with negative semantic words like “Depression” and “Collapse”, their impact on suicidal ideation is even more significant. However, this interaction was not explicitly mentioned in these studies. Therefore, the results of this study further reveal the complex internal relationships and triggering mechanisms between emotional words and suicidal ideation.

Third, the usage frequency, combinations, and information gain of specific words can effectively distinguish between users with and without suicidal tendencies. As shown in Figure 8, in the texts of users with suicidal tendencies, words related to negative emotions and mental states such as “Suicide”, “Cut wrists”, and “Numbness” are used significantly more frequently than in those of non-suicidal users. Moreover, the information gain values of these words in the suicidal context are much higher than in the non-suicidal context, indicating their crucial role in differentiating between the two types of users, which is consistent with the conclusion of previous research [46]. Meanwhile, users with suicidal tendencies have a relatively high frequency of using words related to medical and mental health, such as “Psychologist”, “Side-effect”, and “Anxiety”, suggesting that they may be facing psychological or mental health problems and thus have a relatively high suicide risk. This finding is in line with that of [47]. However, these studies did not consider that their information gain can be used to identify suicidal ideation. In the future, it may be necessary to conduct focused research on some professional psychiatric medications.

In addition, as discovered from Case 9 in Table 6, the DSI-BTCNN model shows a higher probability of identifying suicidal ideation and performs optimally in texts containing more high-frequency words, semantic words, and first-person singular pronouns. This indicates that the model can comprehensively understand the strong suicidal-tendency information conveyed by different POS combinations, as well as the emotional coherence and transitions in the context. It thus gives full play to the advantages of the dual-channel feature extraction structure and the information gain-based fusion mechanism.

### 7.5. Theoretical Contributions

This paper explores the branch-independent architecture design of integrated CNN-LSTM networks, assessing its potential in suicide prevention and introducing innovative theoretical insights.

First, the study introduces a concept of collaborative optimization between sequence modeling and text feature extraction, which synergistically enhances the model’s transparency and interpretability. By acknowledging the hierarchical structure of textual data from words to phrases to sentences, each level is recognized as a source of valuable information [48]. The establishment of independent fork structures allows each channel to operate autonomously in the analysis of suicide-related texts, thereby optimizing the integration of features from multiple perspectives. Based on our comprehensive review, this constitutes the inaugural instance of employing a dual-channel architecture for the detection of suicidal ideation within the context of social media.

Second, this study’s integration of information theory principles, specifically entropy and information gain, into the detection of suicidal ideation on social media represents a significant theoretical advancement. Information theory provides a quantitative framework for understanding and measuring the amount of information contained in data, which is crucial for feature selection and the optimization of model performance [49]. By applying information gain, a concept from information theory, we can identify features that contribute most significantly to the classification of suicidal ideation. This approach ensures the model’s focus is directed towards linguistic nuances that are most indicative of suicidal ideation, enhancing its sensitivity and effectiveness in a critical mental health application.

Third, our study highlights the essential role of data-driven approaches in identifying emotions and suicide intentions, establishing a strong theoretical basis for recognition models using extensive text data from sources like social media. Despite the ethical and privacy concerns that limit the availability of authoritative public datasets, our dataset from Weibo, which is rich in Chinese cultural context, fills a significant gap. It not only provides a valuable resource but also offers new insights for cross-cultural research in the field. Furthermore, our research fosters interdisciplinary collaboration, blending computer science with fields such as psychology and linguistics to develop comprehensive tools for analyzing human language in mental health and beyond, setting a new standard for healthcare and social issue research.

### 7.6. Practical Contributions

The findings of this study lay the groundwork for the development of real-time monitoring and early warning systems, enhancing global capacity for mental health surveillance and intervention. Such systems are particularly instrumental in social media environments where human and medical resources are constrained, enabling the implementation of a proactive suicide ideation alert mechanism.

Upon detection of high-risk indicators, the system is designed to promptly dispatch alerts to pertinent organizations or the families of at-risk individuals, facilitating a swift response in conjunction with established rescue entities, such as the Tree Hole Rescue Organization [50]. This collaborative approach is pivotal in averting suicide crises and saving lives.

Given that suicide is the fourth leading cause of death among adolescents, enhancing social awareness and mental health support for youth is critical [51]. Our research reveals that a high entropy in content signals leads to ambiguity or potential harm, warranting stringent review. Similarly, an elevated entropy in user behavior or content patterns may indicate emotional distress or fraudulent activities, necessitating intervention. Quantifying social media content’s entropy allows for the development of targeted detection models. These models are pivotal for classifying and moderating adolescent posts, safeguarding their mental health. This approach underscores the intersection of social media analytics and public health, meeting the rigorous standards of top-tier scientific research and addressing the urgent needs of young populations.

### 7.7. Limitations

The efficacy of our proposed methodology is substantiated by the results; however, it is not without its constraints. Given the proliferation of unstructured data on social media platforms, including images, videos, and audio [52,53], our research acknowledges the necessity to incorporate multimodal data to enrich the dataset and enhance model performance through data augmentation techniques. Yet, the current landscape of social media research predominantly focuses on image analysis, with scant attention paid to audio and video modalities. The scarcity of publicly available speech and video corpora, compounded by privacy concerns of both users and platforms, presents a formidable challenge in data acquisition. As emphasized in reference [7], future research should be oriented to multimodal data analysis.

In addition, we recognize that emojis typically play an essential role in conveying meaning and emotional intricacies in social media communication. However, as our research is dedicated to the analysis of text features, emojis have not received special consideration. This is an aspect that needs to be taken into account in the future.

Moreover, our model, primarily calibrated within the Chinese cultural context, necessitates further refinement to bolster its cross-cultural and cross-platform adaptability. The model’s generalizability across diverse cultural and social media ecosystems is an area ripe for future investigation.

## 8. Conclusions

This paper introduces a sophisticated deep learning model, DSI-BTCNN, equipped with a dual-channel and parallel multi-kernel neural network, designed to automatically identify suicidal ideation on Weibo. The model leverages the dual-channel architecture to adeptly capture both local textual features and contextual information, thereby unearthing potential suicide-related content and mitigating the discomfort arising from model coupling. Additionally, the meticulously crafted parallel multi-kernel CNN structure broadens the model’s feature extraction capabilities and refines the granularity of phrase extraction, alleviating the strain of architecture overload typically associated with serial CNNs.

To bolster the model’s capacity to discern the significance of each suicide-related cue within microblogs, we employ the information gain-based dynamic feature attention fusion network mechanism. By applying the concept of entropy, this method assesses the contribution of each channel’s features and subsequently optimizes the model’s dynamic response to shifts in the importance of these features. Furthermore, we have meticulously assembled a novel and relatively balanced dataset, specifically tailored for the detection of Chinese microblogs, encompassing 80,000 entries. This dataset serves as an invaluable asset for researchers in computer science, psychology, and education who are engaged in the detection of suicidal ideation. Moreover, a comprehensive suite of experiments conducted on this dataset substantiates the superior performance of our proposed model against established baselines, underscoring its efficacy in the automatic detection of suicidal ideation.

Our research will integrate cutting-edge techniques to explore innovative model architectures, thereby extending the model’s applicability. Additionally, we will investigate the application of information theory to multimodal models and cross-cultural studies, which will deepen our understanding of users’ emotional states. This will be achieved by analyzing the rich information contributed by various cues, including images, videos, audio, and emojis.

## Figures and Tables

**Table 1:** Comparative overview of suicidal ideation detection approaches on social media.

Category	Paper	Year	Method	Data Source	Language	Word Embedding Technique	Evaluation Metrics	Cons and Pros
CNN-based method	Orabi et al. [12]	2018	MultiChannel CNN	Twitter	English	Word2Vec	Acc = 0.83 AUC = 0.92 F1 = 0.82	The method excels in dataset generalization yet struggles with extracting features from extensive texts and subtle contexts.
	Allen et al. [16]	2019	CNN-LIWC	Reddit	English	GloVe	Macro F1 = 0.500	The model’s architecture is commendably efficient, significantly reducing the parameter count required, yet this optimization is effectively constrained to small-scale datasets.
	Yao et al. [13]	2020	CNN	Reddit	English	GloVe FastText	F1 = 0.966	Leveraging Reddit’s metadata boosts the model’s performance via personalized analysis, but it may also heighten sensitivity to outlier instances.
	Li et al. [18]	2023	TCNN-MF-LA	Weibo	Chinese	Word2Vec	Acc = 0.888 P = 0.906	A label association mechanism is proposed to integrate diverse features in the face of missing information, but the model’s training demands substantial investment.
RNN-based method	Alabdulkreem et al. [19]	2021	RNN	Twitter	Arabic	Word2Vec GloVe	Acc = 0.72	This approach is customized for the distinctive features of the Arabic language, providing powerful sequence processing. However, its regional focus restricts broader applicability.
	Matero et al. [20]	2019	Dual RNN	Reddit	English	BERT	Acc = 0.59 F1 = 0.50	The dual RNN structure is strong in dealing with context data but has the problem of vanishing gradients.
	Kancharapu et al. [21]	2022	LSTM	Twitter	English	N/A	Acc = 0.87 F1 = 0.83	Leveraging LSTM capabilities, the sequence processing is enhanced, yet the training is time-consuming and not immune to data bias.
	Deepa J et al. [22]	2023	LSTM	Twitter	English	N/A	Acc = 0.908	The model’s strong sequence processing capabilities help prevent the vanishing of gradients, but it still struggles to fully capture subsequent sentence information.
	Almars [23]	2022	Bi-LSTM-Attention	Twitter	Arabic	Word2Ve	F1 = 0.85	Incorporating a Bi-LSTM structure, the framework boasts potent capabilities for bidirectional sequence data processing, but this comes with a high parameter count and complex tuning requirements.
	Kancharapu et al. [24]	2023	Bi-LSTM-Ensemble	Twitter	English	Word2Vec GloVe	Acc = 0.86	The Bi-LSTM framework, in conjunction with sentiment analysis, effectively detects suicide-related tweets but is ill-suited for large datasets and prone to sensitivity to outliers.
CNN-RNN hybrid-based method	Sawhney et al. [25]	2018	C-LSTM	Reddit Tumblr	English	Word2Vec	Acc = 0.81 F1 = 0.83	C-LSTM excels at capturing local and contextual features, but it faces challenges with text information loss.
	Kour et al. [15]	2022	CNN-BiLSTM	Twitter	English	Tailored Generative Word Embeddings	Acc = 0.94 AUC = 0.95 F1 = 0.95	The model adeptly captures contextual and emotional semantics but is constrained by sentence length limitations.
	Tadesse et al. [26]	2019	LSTM-CNN	Reddit	English	Word2vec	Acc = 0.94 F1 = 0.93	The integration of the two models’ strengths was intended to prevent overfitting, yet it is hampered by insufficient data and potential labeling bias.
	Priyamvada et al. [27]	2023	Stacked CNN-2layers-LSTM	Twitter	English	Word2Vec	Acc = 0.94	The method broadens its feature extraction capabilities, but this expansion entails a large parameter set and demands substantial computing resources.
	Renjith et al. [28]	2022	LSTM-Attention-CNN	Reddit	English	Word2Vec	Acc = 0.90 F1 = 0.93	This approach considers input value relationships and learns hidden text features yet overlooks class imbalance.
	Chadha et al. [29]	2022	ACL	Reddit	English	Word2vec GloVe	Acc = 0.88 F1 = 0.91 P = 0.87	ACL zeroes in on the nuances and key terms of target data but neglects feature beyond social media text.
	Zogan et al. [30]	2022	MDHAN	Twitter	English	GloVe	Acc = 0.895 F1 = 0.89	The multi-level attention network captures detailed user tweet encodings, disregarding topic and sentiment.

Accuracy is denoted as ‘Acc’; ‘P’ stands for precision; ‘F1’ signifies the F1-score, and ‘AUC’ refers to the area under the receiver operating characteristic curve.

**Table 2:** Examples of microblogs with and without suicidal ideation and their likelihood probability for suicidal ideation.

Type	Example	Likelihood Probability for Suicidal Ideation
Suicidal microblogs	I’m seriously considering taking 100 sleeping pills.	99.96%
	I believe the pain is fleeting when inhaling charcoal fumes in the solitude of a bathroom...	97.84%
	Experiencing depression is deeply distressing, and after a month on paroxetine, I feel on the verge of a crisis...	99.99%
	Sandy’s public suicide has left me with a disconcerting inclination to emulate it.	98.94%
Non-Suicidal microblogs	I came to Hong Kong to watch the horse race.	35.03%
	The Forbidden City’s grandeur is so captivating it leaves me breathless!	39.50%
	This photo is so embarrassing, I can’t even look at it!	43.07%
	I have a profound admiration for TVB actresses, including Charmaine Sheh, Ada Choi, and Samantha Ko.	16.62%

**Table 3:** Comparative performance evaluation of word embedding techniques in different models.

Model	Word2Vec					Glove					BERT				
	Acc%	P%	R%	F1%	AUC%	Acc%	P%	R%	F1%	AUC%	Acc%	P%	R%	F1%	AUC%
SVM	72.60	75.49	**66.93**	**70.95**	**81.16**	**72.23**	**83.39**	55.52	66.66	72.71	80.17	**91.28**	66.65	77.05	**91.91**
NB	70.45	73.26	64.39	68.54	78.56	70.02	79.81	53.46	64.03	**82.26**	77.49	90.21	61.60	73.21	87.77
RF	**73.33**	**77.93**	65.10	70.94	79.51	70.44	73.86	**63.27**	**68.16**	78.36	**80.42**	90.88	**67.56**	**77.50**	91.65
CNN	77.50	**90.22**	61.61	73.22	87.77	77.91	86.40	66.16	74.94	86.82	83.91	79.32	**91.65**	85.04	93.80
TextCNN	**82.46**	86.37	**77.08**	**81.46**	**91.32**	**81.25**	**87.44**	**72.97**	**79.55**	**90.88**	**85.27**	81.20	91.06	**85.85**	**94.27**
TextRCNN	80.49	86.48	72.27	78.74	90.05	78.56	86.20	67.99	76.02	88.55	83.51	**91.98**	73.36	81.62	93.52
RNN	76.22	84.72	63.98	72.90	84.26	76.81	**89.06**	61.13	72.50	86.81	83.90	78.77	92.86	85.24	93.12
TextRNN	78.64	88.38	65.94	75.53	90.22	77.96	87.11	65.56	74.81	89.34	84.25	79.33	92.74	85.51	93.49
LSTM	77.75	**89.13**	63.21	73.96	90.11	78.00	85.35	67.59	75.44	86.83	84.46	**79.54**	92.93	85.72	94.02
BiLSTM	**80.58**	87.87	**70.94**	**78.50**	**90.78**	**80.74**	86.46	**72.90**	**79.10**	**90.04**	**84.68**	78.78	**95.81**	**86.46**	**95.41**

Bolded values indicate optimal model performance for each group.

**Table 4:** Performance of different model architectures.

Model	Accuracy/%	Precision/%	Recall/%	F1-Score/%	AUC%
BERT-BiLSTM	84.68	78.78	95.81	86.46	95.41
BERT-TextCNN	85.27	81.20	91.06	85.85	94.27
BERT-BiLSTM-TextCNN	85.76	80.83	**94.25**	87.03	95.28
BERT-TextCNN-BiLSTM	85.98	82.16	92.99	87.24	95.37
DSI-BTCNN	**86.70**	**82.43**	93.38	**87.56**	**95.64**

Bolded values indicate optimal model performance.

**Table 5:** Performance of different numbers of convolution kernels.

Model	Accuracy/%	Precision/%	Recall/%	F1-Score/%	AUC/%	Entropy/%
DSI-BTCNN(1-kernel)	88.78	93.01	85.13	89.07	96.37	80.73
DSI-BTCNN(2-kernels)	87.29	90.52	83.30	86.76	94.79	80.09
DSI-BTCNN(3-kernels)	**89.64**	92.84	**85.90**	**89.24**	**96.50**	**81.75**
DSI-BTCNN(4-kernels)	87.59	90.80	83.66	87.88	95.07	78.99
DSI-BTCNN(5-kernels)	86.67	**93.93**	78.42	85.48	95.92	75.46

Bolded values indicate optimal model performance.

**Table 6:** Examples of microblog text posts for the case study.

Case No.	Text	Probability for Suicidal Ideation in Our Model
1	It’s a **disappointment**. My **memory** has declined somewhat.	92.50%
2	I **lose sleep**. I really want to see a **psychologist**. I feel like I have **severe anxiety**.	96.36%
3	**Bullying** has turned me into an **invalid**, a **lunatic**, a **trash**. I often have **headaches**, **nightmares**, and **low mood**. I’m so miserable.	99.87%
4	I’m so **suffering**. I can’t stop thinking wildly.	92.74%
5	I’m really **suffering**. I really want to **quit my school** and **jump down** from here...	95.98%
6	I’m really **suffering** and **exhausted**. I **sorrow** for myself and I am on the verge of **collapse**. I really **want to die**. **Depression** envelops my world, and the **abyss** of despair seems to swallow me up.	99.90%
7	**I’**m so exhausted. Today **I** was cyberbullied. **They** said **I’**m really not a normal person. **I** want to jump into the river...	99.80%
8	**Zhang Zhi** and **Wang Dong** are often domestically abused. Do **you** think **they** want to jump into the river?	92.29%
9	I’m suffering from **domestic violence**. I’m so **exhausted** and **can’t sleep**. My heart is full of **scars**. The desire to die is getting stronger and stronger. I don’t want to be regarded as a **patient** and **trash**. I seem to want to **cut wrists** and **take medicine**. I want to become a **corpse**. It seems that there is no hope in sight.	99.98%

Bolded words are high-frequency words related to suicidal ideation in our dataset. Since Case 7 and Case 8 explore the relationship between pronouns and the identification of suicidal ideation, only the pronouns are bolded.

## Data Availability

Data are contained within the article. The implementation code is available at: https://github.com/ZoeyYuee/code.git (accessed on 21 January 2025).
